# Bioethics as a Governance Practice

**DOI:** 10.1007/s10728-015-0310-2

**Published:** 2016-01-07

**Authors:** Jonathan Montgomery

**Affiliations:** University College London, ULC Laws, Bentham House, Endsleigh Gardens, London, WC1H 0EG UK

**Keywords:** Bioethics, Governance, UNESCO Declaration, Ethics committees, Legitimation, UK Bioethics

## Abstract

Bioethics can be considered as a topic, an academic discipline (or combination of disciplines), a field of study, an enterprise in persuasion. The historical specificity of the forms bioethics takes is significant, and raises questions about some of these approaches. Bioethics can also be considered as a governance practice, with distinctive institutions and structures. The forms this practice takes are also to a degree country specific, as the paper illustrates by drawing on the author’s UK experience. However, the UNESCO Universal Declaration on Bioethics can provide a starting point for comparisons provided that this does not exclude sensitivity to the socio-political context. Bioethics governance practices are explained by various legitimating narratives. These include response to scandal, the need to restrain irresponsible science, the accommodation of pluralist views, and the resistance to the relativist idea that all opinions count equally in bioethics. Each approach raises interesting questions and shows that bioethics should be studied as a governance practice as a complement to other approaches.

Bioethics is rarely out of the news. A quick look at the BBC website’s health news front page for 1 May 2015 identified a range of stories that most would recognise as raising bioethical issues. They included the first ‘natural’ delivery of a woman who relied on an artificial pancreas, the modification of the DNA of a human embryo, direct-to-consumer testing services (for HIV), malaria vaccine trials, and wheels designed for cat physiotherapy. There can be little doubt that there is something of interest going on, but it is less clear whether there are common threads that link these questions, and, if so, what they are. Should we focus on the use of novel technologies, privileging the modern miracle of the artificial pancreas over the ancient miracle of birth? Or should we stress the human dimension, excluding the feline intervention from the scope of bioethics?

In order for us to address such issues, they need to be identified and framed in a manner that enables us to work towards resolution of the practical dilemmas that they generate. This paper suggests that it is helpful to think about this by exploring the governance practices that have developed to help societies respond to the choices and challenges that arise in the field of bioethics. It does not claim that this should replace other ways of thinking about bioethics, but it does suggest that bioethics governance is an important subject in its own right and that it should supplement other more established perspectives in order to create a fuller picture.

Some mapping of the field of bioethics is necessary if we are to understand the subject matter over which governance is being exercised. However, the boundaries do not need to be precise or fixed. Governance can be established even in the face of disagreement over the inclusion of specific issues within its scope. The paper begins by examining the content of the academic field of bioethics as a subject area for study. It is important for bioethics governance because it speaks to the scope of its jurisdiction. The idea of jurisdiction provides a helpful framework for the consideration of governance questions. As used here, it denotes the processes of marking out the territory in which ‘bioethics’ is accepted to be the most appropriate perspective for considering questions, constituting bioethicists or bioethics bodies as the authoritative decision-makers, and delineating the terms on which this authority is conferred. A jurisdictional perspective enables both descriptive and normative questions to be identified. Thus, we can (and should) consider separately how the jurisdiction has come to be constituted from whether it can be defended as legitimate.

In addition to matters of scope, bioethics governance needs to address questions of working methods and the human resources required. The paper therefore considers briefly aspects of the debate over whether there is a discrete academic or practical discipline of bioethics. There can be little doubt that academics have arranged their work to address the field. Centres, journals and courses are badged under the label of bioethics. The question for the purposes of this paper is whether this has led to a distinctive discipline and methodology. This is linked to the question of whether bioethics as a governance practice should be the province of ‘expert’ bioethicists.

The idea that bioethics can be understood as a practice is not new. As with all academic disciplines, there is a social element to way in which it has developed. In some countries, this has led to the professionalization of bioethics [19], with a degree of governance of the activities of bioethicists [4]. There has also long been a tendency to codify bioethical positions into quasi-legal guidance, extending bioethics from private discussion into a more public and collective process. Parallels can be drawn with other areas of applied ethics, such as business ethics and its manifestation in expectations of corporate social responsibility. However, the extent to which bioethical practices have been consolidated into advisory and regulatory structures (such as ‘ethics committees’) is distinctive. These have a recognized place in global governance mechanisms through the UNESCO Universal Declaration on Bioethics 2005. It is these processes of institutionalization that I suggest constitute the primary forms of bioethics governance. However, although the UNESCO formulation provides a useful framework for discussion, it is not the only manifestation of governance activities.

The UK, for example, deploys a range governance practices beyond the activities specified in the Declaration. The ideas and questions explored in this paper have emerged from the author’s reflections on personal experience of the institutions of bioethics governance in the UK and the primary examples it uses are therefore drawn from there.^1^ One of the arguments of the paper is that attention needs to be given to the socio-political contexts in which bioethics institutions operate if they are to be well understood. Some institutions may look similar but have different roles and scope. Even a superficial comparison between the work of the French Comité Consultatif Nationale d’Ethique and the UK’s Nuffield Council on Bioethics shows that they have significant differences in the topics they have addressed and their ways of working even though they are also in many ways very similar [22, 44].

Bioethics governance should, therefore, be considered in terms of its functions as well as its institutions. In our morally pluralist society, there is considerable divergence of views on many bioethical issues. Various committees, commissions, and authorities have been created to mediate such disagreements, facilitate sufficient consensus to enable public policy choices to be made, maintain public confidence (especially in the responsible use of scientific advances) and reduce the risks that disagreement will manifest itself in social conflict. These responses can be understood as part of an ecosystem of bioethics governance. The aims of this paper are to demonstrate that thinking about bioethics as a governance practice will enhance our understanding of such activities and to draw attention to some features of this approach that merit further consideration, thus sketching an agenda for further study.

## What is Bioethics?

### Bioethics as a Subject

We might seek to define the subject of bioethics by reference to the topics that fall within its scope. The term seems to have emerged in the context of environmental ethics [35, 67, 51, 57], but soon came to be used in relation to medicine and scientific advance. Levine identifies the early agenda pursued by North American scholars in the emerging field of bioethics as comprising research ethics, death and dying, genetics, reproductive technologies and behavioural control [35]. Dunstan, an Anglican theologian active in ethical discussions in the UK in the 1970s selected many similar issues to illustrate his thesis about *The Artifice of Ethics*—a study of the ‘institutions built to support and shelter the frail but precious moral judgments of mankind’—birth control, the use of aborted fetuses in medical research, genetic engineering, IVF, abortion, and euthanasia. He was less clear, however, that these illustrated a specific area of bioethics. His analysis placed these examples alongside questions about business ethics and the conduct of war (covering both the use of lethal force in Northern Ireland and what might now be called ‘weapons of mass destruction’—biological and thermo-nuclear warfare) [16].

Wilson has argued that the identification of bioethics as a separate area for consideration in the UK should be attributed to Ian Kennedy’s 1980 Reith lectures, *The Unmasking of Medicine* [31, 67]. These covered a range of issues that had become medicalized but which Kennedy argued needed to be re-appropriated by society. His opening list contained heart transplants, the definition of death, the treatment of the dying (including prolonging insensate life), the selective treatment of handicapped new born babies, and the treatment of the mentally ill [30]. Thus, he linked UK bioethics to issues in medical practice and its advance.

A flavour of the current scope of the academic literature can be gleaned from the contents of two leading collections. *Bioethics: An Anthology* edited by philosophers Kuhse and Singer contains eighty-one extracts, grouped into sections on abortion, reproductive technologies (including surrogate motherhood, sex selection, embryos as tissue donors and cloning), ‘the new genetics’, life and death, resource allocation, organ donation, experimentation (both human and animal), ethical issues in the practice of health care (mostly relating to aspects of confidentiality and consent), a separate section on issues facing nurses, and finally four pieces on ethicists and ethics committees [34]. This broadly reflects an approach that presents bioethics as addressing choices that are made in and around health services. Methodological issues are generally drawn out within solutions to specific problems rather than presented as of interest in their own right.

Steinbock’s [60] *Oxford Handbook of Bioethics* selects only thirty pieces and takes an approach that focuses less on topics than methods. It opens with a group of essays on theoretical and methodological issues and addresses justice and policy-making before moving to selected essays on the topics of bodies and body parts, end of life, reproduction and cloning, genetics and enhancement, research ethics, and finally justice and global health. This opens up interest in the way in which bioethical matters should be approached, including the possibility that there might be expert ‘bioethicists’; experts who are particularly authoritative guides to the subject. This might manifest itself in the form of a discrete academic discipline, but even this snapshot from two anthologies is enough to remind us that there is sufficient variation in discussion of scope and methods to make the idea that bioethics institutions might be best staffed by expert bioethicists politically controversial [12, 33, 61]. It is perhaps better to think in terms of bioethics being a field or enterprise. As Sheehan and Dunn put it: ‘disciplines are closely tied to methodologies and traditions of thought, whereas what counts as a field is driven by a set of questions’ [59].

### Bioethics as a Discipline?

Harris [25] has argued that the modern understanding of bioethics has two main historical roots. The first is the ethics of the medical profession. He suggests this was more a matter of norms of practice and etiquette than reasoned reflection, although this may not do justice to the richness of the tradition of medical ethics [5, 28]. The second was moral philosophy. It is this that Harris [25] sees as the driver for modern bioethics, suggesting that is essentially a specialist area within applied moral philosophy.^2^ Warnock, also a professional philosopher and a key figure in UK bioethics, pursued a slightly different dynamic, resisting the idea that philosophical expertise should determine public policy while welcoming the role of bioethics in rescuing British philosophical ethics from an analytical dead-end by replacing the focus on the logic of ethical discourse with an interest in substantive questions [67]. These claims raise questions about the disciplinary nature of bioethics. Conceptual work is needed to define the scope of bioethics, and refine its methods so as to enable poor and robust work to be distinguished. However, its connection to policy-making needs further explanation.

Further, the way in which bioethical work is organised and funded has a significant impact on what counts as bioethics. In the USA in the 1970s, bioethics emerged as a new academic and practical discipline through the creation of institutions. The Hastings Center, originally the Institute of Society, Ethics and the Life Sciences was established in 1969 [9, 35]. The Kennedy Institute of Ethics at Georgetown was established in 1971 [35].^3^ Many more followed as bioethics took root in the academy. The American Society for Bioethics and Humanities now has over 1800 members.^4^ It is hard, therefore, to resist the conclusion that bioethics is an area for academic activity. However, it does not follow that there is a discrete academic discipline with a distinctive set of concerns, conceptual tools and methodologies.

Context is crucial. Rothman argues that there was a very specific congruence of political forces that made the context in America particularly hospitable to the emerging thinking on bioethics. He cites the civil rights movement’s wider challenges to power and authority, including anti-discrimination provisions that became applied to neonatal care decisions, the Patient Bill of Rights (formally developed by the America Hospitals Association in 1973), and the development of the right of privacy in the courts in the key medical case of *Roe v Wade* on abortion. The particular manifestation of bioethics suited the *zeitgeist*.

The fit between the movement and the times was perfect. Just when courts were defining an expanded right to privacy, the bioethicists were emphasising the principle of autonomy, and the two meshed neatly; judges provided a legal basis and bioethicists, a philosophical basis for empowering the patient. Indeed, just when movements on behalf of a variety of minorities were advancing their claims, the bioethicists were defending another group that appeared powerless - patients. All these advocates were siding with the individual against the constituted authority; in their powerlessness, patients seemed at one with women, inmates, homosexuals, tenants in public housing, welfare recipients, and students, who were all attempting to limit the discretionary authority of professionals [57:245].

On this view, the discipline of bioethics is aligned with philosophy, political theory and human rights law. In terms of the disciplinary power, bioethics is characterised as competing with medicine for jurisdiction.

Evans [19] provides a sociological account of the history of US bioethics that also concentrates on the struggle for jurisdiction, but characterizes it as being between science and theology. He describes the transmutation of the work of theologians into bioethics in the form of ‘principlism’, which he sees as the distinctive methodology on which the claim of bioethics to be a ‘discipline’ is based. In line with the political philosophy of Rawls, and in particular his idea of ‘public reason’ [54], Evans argues that this enabled bioethics to claim a degree of neutrality between substantive approaches. He notes Engelhardt’s position that bioethics could develop a ‘moral lingua franca’ without ‘endorsing a particular moral vision’ [18:ix].

These two rather different approaches share the insight that the nature of argumentation in bioethics is shaped by the circumstances in which it emerges. This should make us cautious on putting too much weight on the conception of bioethics as a discipline. The intellectual approaches that have driven bioethics in mainland Europe have been different, with more focus on personhood, the virtues of patient-professional relationships, solidarity and human dignity [58]. The USA has been dominated by Beauchamp and Childress’s four principles of autonomy, non-maleficence, beneficence and justice [6].

Both approaches can be found in the UK. However, the dominant view in Britain in the 1970s was that medical ethics assisted doctors to make better decisions, and that this was contrasted with the ‘American trend’ of bioethics in which outsiders had assumed the role of ‘society’s conscience’ on issues previously entrusted to doctors [66]. This focus on supporting health professionals to make ethically informed decisions, on the assumption that they adhere to a system of moral values has continued to underpin much of legal regulation in the UK [38, 39, 42]. This has not prevented the emergence of a significant ‘bioethics industry’, but it has taken the form of practices for public governance.

British work on bioethics did not begin with the creation of new academic centres, but by working within the established professional institutions, in what Wilson describes as ‘club regulation’ [67:ch 2]. This is less clearly rooted in disciplinary competition. He sees the emergence of UK Bioethics as an aspect of the ascendancy of the Audit Society, in which social institutions were constrained and controlled by measurement and oversight [67:ch 3]. He argues that although Kennedy’s Reith lectures adapted an intellectual discipline of bioethics from the US academic tradition, it took root because of its congruence with the Thatcherite programme to break down the power of the traditional professions. Wilson sees the 1980s and 1990s as the ‘high water-mark’ of bioethics. Prior to these decades, what is now known as bioethics was a collaboration of the professional establishments of church, medicine and law. During those decades, the professions were drawn into conflict and competition with each other by wider social processes. The emergence of bioethics was one of the ways in which those conflicts played out. Wilson cites the critique of the Audit Society by Baroness Onora O’Neill [46] in the Reith lectures for 2002 as a further watershed as the general retreat from big government manifested itself in the dismantling of the instruments of bioethics governance. He suggests that ‘today, neither the government, nor many bioethicists, share Kennedy’s belief that oversight is the best way to ensure public accountability [65].’

Bioethics in the UK thus moved more quickly into governance practices than academic ones and, perhaps as a result, has not reached the same disciplinary definition as seen in the USA. Indeed, in the UK, few people see themselves as professional bioethicists [10]. Rather, Onora O’Neill has suggested, bioethics should be seen as a communal practice. It ‘is not a discipline’, but instead provides ‘a meeting ground for a number of disciplines, discourses and organizations concerned with ethical, legal and social questions raised by advances in medicine, science and technology’ [47:1]. This identification of bioethics as a field of study, to which many disciplines can contribute suggests the need for mechanisms for co-ordination of activity.

### Bioethics as an Enterprise?

This co-ordination is often directed at a particular kind of purpose. One of the characteristics of contemporary bioethics is that it has taken a ‘public’ turn, in which it ‘constitutes a resource for the formation of public policy which impacts upon the social world [52:8].’ Sheehan and Dunn have described this as a requirement of ‘practicality’, suggesting that it counts against a bioethical argument that it could not be implemented.

a piece of research or public activity is not correctly defined as bioethics unless it aims at actually convincing people to act differently or to change policy because of the arguments and answers that the bioethicist provides [59:58].

Bioethics must be ‘sensitive to the realities of political contingencies and institutional constraints’ [59:58], and

It is an entirely appropriate response to an argument in bioethics that is impractical, or, for example, that its actual implementation would not succeed because the argument fails to consider relevant contextual features of the relevant situations [59:59].

They describe bioethics as concerned with primary ‘ought’ questions (e.g. the best policy on abortion), to which a series of secondary questions are relevant because of the practicality requirement. These include ‘the nature and functioning of the regulatory system.’

However, the issues of practicality perhaps become the primary, rather than secondary questions when bioethics is considered as a public facing enterprise. The starting point for addressing the ‘ought’ questions is the available practical options and a substantive position is already implicit within the status quo. The issue is about whether there is a case for change. The onus of proof lies on those who propose reform and the burden of proof requires a sufficiently compelling argument to justify investing the energy necessary to overcome the forces of inertia. The playing field is not an even one, but the unevenness is entirely contextual. The status quo may be different in different places in relation to the same question. Reform proposals developed by bioethicists should therefore be understood in their specific historical, political and social contexts [43].

Thinking about bioethics as an enterprise, aimed at public persuasion and impact, enables us to focus on dimensions that are marginal to the normative tasks of the version of the discipline that sees itself as rooted in applied moral philosophy. We should think not so much about what bioethics ‘is’ as about what it does. We should be concerned to understand the nature of bioethics as a Foucauldian ‘discipline’, a discursive technology of social control [56], and look for a normative framework for critique that is sensitive to the way in which bioethics asserts its jurisdiction in matters of public significance, not merely private morality. We should also be concerned to study the institutions by which society governs matters of bioethical significance. Dunstan describes a societal activity in which ‘the moralist, having seen his (sic) vision, or arrived at his position, must weave his insight into the fabric of society by creating an institution in which to embody it’ [16:4]. We must consider the structures and functions of bioethics governance.

## Bioethics as a Governance Practice

### Typologies of Governance Practices

The examination of institutions shifts the focus of the practice of bioethics from an intellectual enterprise to a governance one. Duwell has described an *institutionalisation* of bioethics as a response to a mixture of demands from clinicians for support, emerging public concerns, (including those about technological advances and also scandalous behaviour), and the changing political contexts in which long-standing questions about the value of life were debated and translated into principles and rules to guide public life. He draws attention to three types of committee. First, those formed to advise political institutions (concerned with ethical reflection on emerging problems). Second, those to provide assurance that established ethical principles have been observed (such as research ethics committees). Third, those devised to support individual decision-making in particular cases (typically described as ‘clinical’ ethics committees). Each of these aims to provide support on bioethical issues, but in different ways and for different purposes. The first is aligned with existing political or professional authority (such as governments, hospitals) and offers advice on how to exercise it. The second rarely engages directly in ethical reflection, but is concerned with ensuring compliance with established standards. The third is concerned with specific cases and Duwell suggests that it serves to ‘create a space within the clinical praxis in which conflict situations can be dealt with transparently with regard to both argumentation and procedure’ [17:2–5].

A similar typology can be derived from the UNESCO Universal Declaration on Bioethics (2005), which adds a fourth category about wider public discussion (one recognized by Duwell but not considered to be institutionalized). Article 19 of the Declaration states:

Independent, multidisciplinary and pluralist ethics committees should be established, promoted and supported at the appropriate level in order to:

(a) assess the relevant ethical, legal, scientific and social issues related to research projects involving human beings;

(b) provide advice on ethical problems in clinical settings;

(c) assess scientific and technological developments, formulate recommendations and contribute to the preparation of guidelines on issues within the scope of this Declaration;

(d) foster debate, education and public awareness of, and engagement in, bioethics.

This international instrument has legitimated both a capacity building programme,^5^ and also the comparison and critique of the bioethics governance structures in different states [48, 62, 63]. Even though the forms that bioethical governance practices take must be understood as generated within a specific historical context, comparisons and discussions can only proceed with a schematic analysis of some sort. However, we should not limit the conception of governance processes to this schema as the tools that are used in bioethics governance are more contingent and diverse than the UNESCO Declaration suggests.

### The Historical Contingency of Bioethics Governance

Understanding this requires historical consideration of how bioethics became institutionalised into the specific forms of committees, regulatory bodies and commissions. Jonsen has documented the transition from the 1960s as a ‘decade of conferences’, in which scientists from across the world came together to discuss the emerging possibilities, into the 1970s when bioethics became the province of Government commissions [29]. The emergence of these earliest governance bodies seems more nationally determined. Rothman’s study of how medical ethics became detached from the internal morality of the profession traces the tousles in the US Congress that led to the establishment of National Commissions [57:ch9]. He shows how professional resistance to external scrutiny frustrated politicians and contributed to the creation of Commissions on which medics were in a minority. Professional witnesses denied the relevance of the concerns put to them, suggested that outsiders lacked competence in these matters, and implied that their appearances before the committee were a waste of their valuable time.

In this context, the transfer of power and authority in bioethics from the medical profession to ‘strangers’ by the creation of commissions to deliberate on issues of principle can be seen to flow from the resistance of the profession to the legitimacy of public debate, and its denial of political authority in the area. This was extended by the Superior Court of New Jersey in the *Quinlan* case, which suggested the use of committees to oversee individual clinical decisions as a way of addressing perceptions that doctors were faced with conflicting interests and to provide protection from civil and criminal liability [27]. Despite the fact that this was not a decision that was legally binding in other states, the decision was followed by increased formality within hospitals for oversight of clinical decisions; an Optimum Care Committee was established at Massachusetts General Hospital, formal guidelines began to be drawn up and reference to court increased. Lawyers began to play a prominent role [57:229–235].

Robert Baker attributes the particular shape of the development of bioethics in the USA less to the arrogance of doctors in respect of ethical issues than to their complacency:

organized medicine’s laissez-faire abandonment of medical ethics created a void in the marketplace of ideas and a vacuum of moral authority. To fill this void, legislators, bureaucrats, the courts, and American society generally sought ideas and invested moral authority elsewhere, ultimately finding it in an oddball collection of lumpen intellentsia who were soon valorized as ethics experts or “bioethicists” [5:279].

He points out that in Europe, organized medicine never abandoned its jurisdiction over ethics. Consequently, bioethics developed as a collaborative not antagonistic enterprise.

Thus, the institutions of bioethics governance do not play precisely the same roles in different societies. We have already noted Wilson’s description of early British bioethics as a system ‘club regulation’. This collaborative approach continued with the emergence of institutions of bioethics governance. In the UK, the medical profession was not forced into developing such bodies, but took a lead. It moved more quickly than Government to establish a body to provide ethical oversight in the context of assisted reproductive technologies. A Voluntary (later renamed Interim) Licensing Authority was established soon after the publication of the Warnock report in 1984 [55], and broadly played the same role as was subsequently taken on by the Human Fertilisation and Embryology Authority from 1991 when the legislation came into force. The doctors’ trade union, the British Medical Association developed a Handbook of Medical Ethics that promoted a focus on raising expectations that doctors should meet ethical standards in advance of the regulator’s interest which for many decades was on misconduct rather than good practice [41:42–44]. British bioethics has been less confrontational than its US equivalent and the history and functions of its institutions need to be considered with that in mind.

However, we should not to develop too narrow a conception of governance processes. There is more to governance than the constitutions of committees. It is also important to recognise the messiness of the connection between the institutions actually created and the stylized explanations of their purpose. These points can be drawn out of a brief discussion of some of the functions of bioethics governance.

## Functions of Bioethics Governance

Evans’ [19] sociological perspective on bioethics identifies four jurisdictional spaces that might be inhabited by bioethicists. These concern health care ethics consultations, research bioethics, public policy bioethics, and cultural bioethics. Evans’ account stresses the importance of understanding the ‘jurisdiction givers’ who provide access to these spaces. He suggests that the possibility of the professionalization of bioethics in the USA, and also the principlist form it took, were a consequence of the government officials becoming jurisdiction givers. Their expectations were for an abstract body of knowledge that could claim to provide a common morality in a pluralist society. As bioethics adopted this form it gained jurisdiction. Evans argues that the status of professional bioethics is waning in the USA because both government and the media (as gatekeepers to cultural bioethics) are turning directly to the spokespeople for partisan social movements rather than looking to bioethicists to mediate the opposing arguments.

This section of the paper considers four justificatory narratives that provide plausible explanations for governance activities, and therefore serve to legitimate it. These are the response to scandalous activity, the imperative to address public concerns about potentially irresponsible scientific advance, the need to resolve deep disagreements about bioethical matters in a pluralist society, and the desire to delineate public bioethics from other political issues. These narratives are not the only ones that might be considered, but they are sufficient to show that thinking about bioethics as a governance practice raises distinctive questions that merit further study.

### Bioethics Governance as a Response to Scandal: The Case of Research Governance

One of the dominant narratives that serves to drive bioethics governance is that it is a necessary response scandals of professional immorality. This is perhaps most easily seen in relation to the governance of medical research. On this account, the abuse of human research subjects leads to regulatory interventions to control researchers and protect participants. While doubts have been raised about the historical accuracy of this explanation in respect of specific regulatory developments [26], there is little doubt that it is seen by many as the explanation for the need for governance.

Even if this account of the origins of research governance is more myth than history, it enables us to connect the rationale with an appropriate framework of critique. Given that scandal is generated by events that are considered to transgress norms (even if those norms were never explicit), a successful governance framework will address both the occurrence of such events and the management of expectations in order to sustain public trust and confidence. This need not be seen as an exercise in external control. The research community has an interest in good governance provided that it serves to maintain the social licence that it requires for its work [15].

So far as the tools of bioethics governance are concerned, three dimensions of the regulation of health research are worthy of mention. The first concerns the codification of principles. Here, the response to the abuses of Nazi medicine that were revealed in the War Trials is often regarded as pivotal. The ‘Nuremberg Principles’ stressed the primacy of individual rights over the advancement of science and the importance of informed consent [1, 23]. The World Medical Association took this approach forward in the Declaration of Helsinki, first adopted in 1964 and amended for the seventh time in 2013 [69]. It is important not to claim too much for this component of bioethics governance. Germany was the first country to pass laws protecting research subjects, so Nazi medicine was not pursued in ignorance of the requirements [20:106, 23]. Nevertheless, standards are an important tool. They make it clear to researchers what is expected of them, hopefully providing a guide to their conduct but also enabling them to be called to account. They can also be protective, offering a means to explain to the public that they are acting ethically. Committees charged with scrutinizing research cannot be identified as ‘ethics’ committees without some set of principles to define ethical issues. In the absence of such standards, even if they are gatekeepers whose permission is required before research takes place, they not are not engaged with bioethics governance but with other types of question such as scientific review, prioritization of resources or reputation management.

Principles may be essential to the creation of an ethical governance system, but they are not sufficient. As the exposés of Beecher in the USA [7], and Pappworth in the UK [49, 50], showed significant numbers of medical research studies were (in various different ways) unethical long after the Nuremberg Principles had been promulgated. This was more a professional than public scandal, although Beecher’s criticisms were neglected until amplified by journalists. Both came from within the medical profession not outside it. So too did the most visible governance mechanism in medical research, the Institutional Review Board or Research Ethics Committee. This was promoted by the US Surgeon General in 1966 on prompting by the US National Institutes of Health (which had had some sort of ethical review since 1953) [26:333] and extended as a regulatory requirement by the Belmont Report in 1979 [57:ch5]. As Adam Hedgecoe has shown, the history of RECs in the UK is similarly bottom up [26]. It began in hospitals seeking US funding. It was picked up pragmatically by the Royal College of Physicians in the form of a Committee on the Ethical Supervision on Clinical Investigations in Institutions, which reported in 1967. Committees were required administratively by the Department of Health in its *Red Book* of 1991 [13]. They only became a legal requirement in 2004 following the implementation of the EU’s clinical trials directive [37]. Prior review by gatekeeping committees is now an established part of the governance structure for medical experimentation to provide assurance that studies are designed in accordance with the ethical principles previously established.

In itself, this does nothing to ensure that studies are well conducted and following a research scandal in North Staffordshire [24], the UK National Health Service put in place a further dimension of the governance, known as the Research Governance Framework for Health and Social Care [14]. This incorporated the features already described; a restatement of principles (the primacy of the rights of participants, informed consent, confidentiality and data protection) and the requirement of ethics review. However, it went further and specified the separate responsibilities for the conduct of the trial that lay with (a) principal investigators, (b) research sponsors, and (c) the organisations which employ the researchers. These clarifications have enabled accountability for breaches in the conduct of research, monitored by the Health Research Authority, although regulatory action in this area remains rare.

Thus, we have an archetype of a bioethics governance structure; codified ethical standards, licensing through ethical review and oversight/accountability through research governance. In origin, this emerged from within the professions and serves to protect its reputation. Its continuation is typically justified by reference to a series of historical scandals against whose recurrence the governance system is claimed to provide protection for participants.

### Bioethics Governance as the Restraint of Irresponsible Science

A second explanatory narrative for the need for bioethics governance lies in fears about scientific advance. In this story, science is portrayed as being driven by a technological imperative, doing things because it can, without regard for whether it should. This was one of the planks of the argument put forward by Kennedy for the establishment of national a bioethics commission on the USA model in the 1980s [32]. It is this aspect of bioethics governance that lay behind the creation of the Nuffield Council on Bioethics in 1991. Its terms of reference, as yet unchanged, require it:

To identify and define ethical questions raised by recent advances in biological and medical research in order to respond to, and to anticipate, public concern;

To make arrangements for examining and reporting on such questions with a view to promoting public understanding and discussion;

It is also charged with publishing reports on these matters and making representations to appropriate regulatory or other bodies. The working assumption is that scientific advances are a matter of public concern that needs to be allayed.

As with the evolution of research governance, it would be wrong to see the creation of commissions charged with considering the implications of scientific advance as external regulation driven by lay people or even as necessarily resisted by scientists. Warnock, another advocate of a national commission, suggested that it would be a counter-balance to

an almost medieval obscurantism… a hostility to science based on vague thoughts that there are some things that we should not know, but based more than anything on fear and ignorance…. After the last war there was there was a cliché to the effect that man’s scientific knowledge has outstripped his moral sense. At that time it was uttered in the context of the physical sciences. The bomb had, rightly, frightened us all. Now the same cliché is more and more to be heard in the context of the biological sciences. We must take it seriously. Only within an ethical framework widely seen to be secure and sensible can we continue, as we must, to push back the frontiers of science [64].

Although the source of public concern is different, bioethics governance is once again a mechanism for enabling public confidence to be maintained.

It is a moot point whether ‘science’ is a single thing about which concerns are raised, or whether the need for governance arises differently in relation to distinct issues. The Nuffield Council on Bioethics, as a non-government body, does not precisely reflect the expectations of the UNESCO Declaration. It is, however, the only British body with an overarching remit for keeping new bioethical issues under review. More characteristically, the tendency of the UK has been to address bioethical issues through specialist institutions rather than a generic one. Thus, issues in assisted human reproduction are on the agendas of many national bioethics commissions but in the UK they have been addressed by sector specific means. The Warnock Committee reported on the policy in 1984 [55], leading to the creation of the sector regulator the Human Fertilisation and Embryology Authority. Questions around organ donation were explored by the Redfern Committee into a scandal at Alder Hey, the Retained Organ Commission, a sector regulator (The Human Tissue Authority), a Parliamentary Select Committee and an Organ Donation Task Force. Non-Government investigations into organ donation have included reports from the King’s Fund, the Nuffield Council and from the BMA. The UK’s preparation for pandemics has included a Committee on the Ethics of Pandemic Influenza. Some of the issues around the biodata being gathered by Genomics England have been addressed by the ad hoc route of asking a trusted bioethicist to convene a group of people to help draft a letter of advice to the Chief Medical Officer. Parliamentary Select Committees have examined a number of other bioethical questions generated by scientific advances, including regenerative medicine, mitochondrial DNA donation, and the use of biodata.

It is also clear that there are important bioethical issues that are perceived to give rise to governance challenges that are not driven by scientific advance but by clashes of values. While interest in bioethics governance can be promoted by ‘morally disruptive’ technologies [5], it is not necessary to have new technologies for bioethical controversy to arise. This can be seen in relation to debates over abortion, where technological advances (such as the move to medical rather than surgical methods) provide the opportunity to revisit fundamental conflicts over the status of the human fetus rather than generate new ethical arguments. It can also be seen in deliberations over end of life care, where the arguments are well known but the solutions remain hotly contested. In the UK, the private member’s bill process has led to full Parliamentary debates on assisted dying in both Houses, but this has not resolved the matter.^6^ We need therefore to consider further explanations of the role and function of bioethics governance processes.

### Governance as a Response to Pluralism

A starting point would be the fact of deep disagreement; a problem of moral pluralism [18]. This creates a challenge for bioethics governance mechanisms to achieve a sufficient degree of closure to enable health and research systems to function. This will clearly be a key feature of clinical ethics support, in which the patient’s position will often dictate a timescale for decisions to be taken. In the policy context, decisions may also need to be taken within specific time frames. There is no neutral position in the face of controversy about, or professional and public demand to be permitted to use, new (or indeed established) technologies. A decision to sustain the status quo needs justification just as much as one to reform the position. The decision cannot, therefore, be ducked. Amongst the functions of bioethics governance is to legitimate such decisions.

Sometimes, this might be a form of closure that claims to have resolved a substantive dispute and answered the concerns. In other circumstances, however, the position may be reached on a temporary or provisional basis, so that pressing policy decisions can be made but revisited at a later date. Here, it might be better to describe the governance process as one whereby jurisdictional distinctions are created that allocate the decisions to particular decision-making processes. Under such an approach, an issue is regarded as closed in certain fora, and the settling of controversy is deferred or diverted to a different forum. Thus, pluralism is acknowledged in a way that balances the desire for public debate with the need to facilitate research and clinical care.

An illustration can be found in the terms of the Abortion Act 1967, which sets out conditions when termination of pregnancy is permissible. For the purposes of individual clinical decisions this defines the ethical questions, balancing women’s rights with other values. The responsibility for assessing whether those conditions are met is allocated to two medical practitioners. Accountability is limited to the scrutiny of their good faith and does not extend to the substantive judgment itself. Controversy remains about the legitimacy of this settlement but it is deferred away from the clinic to Parliament, managerial oversight and political campaigning. In such fora, the issues are far from resolved.

A further aspect of jurisdictional deferral can be seen in the Human Fertilisation and Embryology Act’s tiers of authority to create norms [40]. Some matters are determined by Parliament, some by the HFEA through its licensing conditions or Code of Practice, and some by clinics or individual clinicians. This distribution of authority is a key technique of bioethics governance. These decision-makers remain accountable through Parliament and in the courts. Without this, the jurisdictional settlement would become unstable but with sufficient accountability it can handle the challenges of balancing competing values in society and enable decisions on bioethical issues to be implemented. Separating out these tiers of norm-making powers enables the differentiation of who should be involved in which types of activities. It seems legitimate for the HFEA to exclude from the licensing committee those who are opposed on principle, in order to ensure that the legislation is applied and not undermined. However, a governance structure that completely prevented the possibility of dispute over such issues would soon lose its legitimacy.

If bioethics governance is to be an effective response to the challenge of moral pluralism an account is therefore required of the ways its processes for mediating between conflicting factions demonstrate sufficient respect for differences to justify acting on conclusions reached [8]. This might involve some appeal to the representativeness of the membership of governance bodies. Thus, the constitution of some national ethics committees requires that membership includes a range of characteristics that reflect the diversity of the populations. The Belgian Advisory Committee on Bioethics has its origins in a co-operation agreement of 15 January 1993 signed by the federal Government, the French-speaking Community, the Dutch-speaking Community, the German-speaking Community and the Joint Commission for Community Matters. It has 35 voting members, representing various constituencies, each of which must evenly balance French and Dutch speaking members. Each of member has an alternate to ensure that their perspective is not lost if they are absent.^7^

It seems clear that the fact of pluralism is one of the important contexts in which the institutions of bioethics governance function. However, it does not explain the creation of bodies specifically concerned with bioethics rather than more general approaches to public debate and democratic decision-making. The observation by Evans that in the USA, the gatekeepers have turned away from inviting professional bioethicists to dominate the jurisdictional spaces in favour of partisan social movements draws attention to the need to identify a justificatory narrative for bioethics governance that makes a substantive ethical contribution to the ways in which pluralism is acknowledged.

### Governance as a Response to Relativism

A fourth sustaining narrative for bioethics governance concerns its claim to move beyond a relativist assumption that all ethical opinions are entitled to equal respect. It needs to be more than just counting votes if it can claim to be worthy of separate consideration beyond other democratic mechanisms. Issues might be resolved by plebiscite, counting votes, as in the decision of Oregon to permit physician assisted suicide [58]. It has been argued that the governance of new health technologies in the European Union has been determined more by regulation of markets, based on managing risk and respecting human rights, than by ethics [21]. Bioethics governance seems to be based on the idea that there is something sufficiently distinctive about the topics within its scope to render these mechanisms unsatisfactory. It aims for substantive ethical argument to drive decision-making. Market approaches make such arguments exceptional, reasons to restrict the normal economic freedoms. Plebiscites privilege the quantity of support rather than its basis. An opinion counts even if it is rationally indefensible or based on scientific error.

The response to challenge relativism takes one of two main forms. The first is substantive and takes the form of a search for common principles that can then be implemented through the governance institutions. In this model, the relationship between the committees and the ethical debates may be ambiguous and questions arise as to how far they should be concerned with substantive ethics and how far with ensuring compliance with standards of which they are stewards not creators [2, 36]. The response to relativism lies in the robustness of the principles rather than the enforcement processes.

We have already seen how the emergence of principlism in the USA to take this role can be linked to the Rawlsian idea of ‘public reason’. The essence of this approach is the use of concepts that can provide a common currency for debate, even though they will be supported by participants for different (and sometimes contradictory) reasons, and do not involve judging the morality of others: ‘it neither criticizes nor attacks any comprehensive doctrine, religious or nonreligious, except insofar as that doctrine is incompatible with the essentials of public reason and a democratic polity’ [54].

In the context of clinical ethics, there are manifestations of the public reason approach in the form of professional guidance, consensus statements and regulatory requirements for maintaining a licence to practise. The study of bioethics governance might take the form of reviewing such documentary manifestations as evidence of the content of bioethical public reason.

In relation to the wider functions of bioethics in support of public policy, international conventions can be seen as a similar response to the challenges of relativism through the establishment of a foundation of common principles. The UNESCO Declaration has already been noted. The Council of Europe’s Oviedo Convention provides another example. Developed within the human rights paradigm, it pronounces on issues that are firmly within the scope of academic bioethics and subsequent protocols have extended its reach further. Whether the process is seen as intellectually satisfactory or merely an exercise in politics will determine whether this turn to ‘public reason’ provides a satisfactory response to the problem of relativism [3] and requires consideration of the logic of public ethics [43, 68].

There is a second approach that seeks to address the challenge of moving from disagreement to some normative framework without simply accepting the validity of different views in our pluralist societies. Here the focus is not on the premises and conclusions of public reason, but upon the character of the processes that are thought in themselves to provide legitimacy for the positions reached. The Nuffield Council on Bioethics has adopted a position of this type. A stock take of its work, by Chan and Harris in 2006, suggested that it might have incrementally developed something a little like ‘Nuffield Council Ethical Framework for Bioethics’ [11]. They identified a number of principles that seemed to be drawn upon, although not always consistently; avoidance of harm, the duty of beneficence, respect for persons and autonomy, justice and just resource allocation, informed consent, confidentiality and privacy, respect for dignity, and naturalness.

Since then, the Council has committed itself to a different legitimation narrative based on the procedural aspects of public reasoning rather than its conceptual content. In response to the fact of pluralism, it has committed to a principle of ‘inclusiveness: no single view or approach to bioethics should be favoured, and the expression of all views should be encouraged and welcomed [45].’ On this basis legitimacy can be drawn partly from the fact that no one has been excluded from the debate. In response to the problem of relativism, it has asserted the importance of applying to all arguments ‘tests of coherence and rationality’ in a rigorous way, ‘based on the best evidence available, and supported by careful and comprehensive analysis’ [45]. Here the approach seeks to distinguish the resolution of disagreement through compromise and negotiation from bioethics by characterizing it as an evidence-based argumentative activity in which participants must justify and not merely assert their positions.

## Conclusions

The approach adopted by the Nuffield Council could be little more than a particular manifestation of the principles of deliberative democracy. However, this is where it becomes necessary to reflect on the interrelationship between the senses of bioethics that were explored in the earlier sections of this piece. It is a deliberative process, but it is overseen by a group of people selected for knowledge and interest in the disciplines that the Council perceives relevant to contribute to the field of bioethics. The idea of bioethics as a discipline is rejected in favour of an understanding of a field of inquiry. However, the activities of the Council are very much of the nature of an expert enterprise in policy engagement. The Council is influential in policy fora, but has no formal authority. It is therefore reliant on implementation structures being in place for bioethics governance. So, bioethics is all of the things that have been discussed. However, its nature as a governance practice is an important dimension of the ecology of bioethics.

Recognition of the value of seeing bioethics as a governance practice, raises areas for enquiry that can too easily be overlooked. First, the legitimacy of its institutions to operate in the public sphere needs more explanation and justification than it currently demonstrates. This will include consideration of the people who are involved; how they are selected; the nature of the authority that they exercise; the processes by which positions are reached; the efficiency, proportionality and effectiveness of the accountability mechanisms that can be invoked. Second, further elaboration of the forms of institutionalization of bioethics is needed. Third, there is scope for study of the literary forms of bioethics governance; such as opinions, reports, guidelines, consensus statements. The forms of dissemination of public ethics are different to those of the academy and these differences are worthy of examination. Studying bioethics as a governance practice focusses more on *who* does things, *how* and *why* they do them, than in *wha*t they study and what they conclude. It should not supersede other approaches to understanding bioethics, but it should complement them.

## Footnotes

1 The author is currently Chair of the Nuffield Council on Bioethics, and also of England’s Health Research Authority. Previously, I was Chair of the Human Genetics Commission (2009–12), a Working Party on a Strategy for Brain Tissue Banking for the UK Clinical Research Collaboration (2007–8) and the Southampton and South West Hampshire Joint Research Ethics Committee (1998–9). My memberships of UK committees engaged in bioethics governance have included the Committee on the Ethical Aspects of Pandemic Influenza 2006-, the Human Genomics Strategy Group 2010–12, the Organ Donation Taskforce 2008–10, and the Medical Ethics Committee of the British Medical Association (2003–8). The views expressed in this paper are personal and do not represent the positions of any of these bodies or committees. This paper is a revised version of the 2015 Annual Lecture of the Centre for Health Ethics and Law at Southampton Law School, held in conjunction with Health Care Analysis. I am grateful for the comments of the members of the Centre and the two anonymous reviewers for the journal. I take full responsibility for the errors that remain.

2 See Wilson [67] for discussion of how this philosophical assumption came to dominate.

3 Originally established as the Joseph and Rose Kennedy Center for the Study of Human Reproduction and Bioethics.

4 www.asbh.org (last accessed 23 October 2015).

5 Assisting Bioethics Committees, see http://www.unesco.org/new/en/social-and-human-sciences/themes/bioethics/assisting-bioethics-committees/ (last accessed 23 October 2015).

6 Assisted Dying Bill 2014, Assisted Dying Bill (No 2) 2015.

7 http://www.health.belgium.be/eportal/Healthcare/Consultativebodies/Commitees/Bioethics/Mission,composition,functionin/index.htm#.Vh7DEs9dGUk (last accessed 14 October 2015).
